# Predictors of time-to-death on children in Tigray regional state, Ethiopia: a retrospective cross sectional study

**DOI:** 10.1186/s13690-021-00635-y

**Published:** 2021-06-25

**Authors:** Gebru Gebremeskel Gebrerufael, Bsrat Tesfay Hagos

**Affiliations:** 1 Department of Statistics, College of Natural Science, Adigrat University, P.O.Box.50, Adigrat, Ethiopia; 2 Department of Statistics, College of Natural Science, Mekelle University, P.O.Box.231, Mekelle, Ethiopia

**Keywords:** Children, Time-to-death, Tigray regional state, Predictors, Cox regression model

## Abstract

**Background:**

Although, the clinical and socioeconomic condition of Tigray Regional State mothers has been improved along with the decline in the child death rate. However, children’s death rate is still one of the main community serious challenging issues of public health concern. Thus, the main objective of this current investigation was to identify the major predictor factors for short time-to-death in Children in the Tigray Regional State.

**Methods:**

The study used a secondary data with cross-sectional study design.

The information gathered was from 1018 childbirths 5 years prior to the survey. Independent variables such as mother’s demographic variables, child demographic variables, healthiness and environmental factors were considered major hazard predictors of children’s short time-to-death rate. This current investigation used bivariable and multivariable Cox regression model analysis to identify the major statistically significant associations with children’s time-to-death rate.

**Results:**

One thousand eighteen children under 5 years of age were included in the study.

Of them, 50% of the children were males, and the median survival time-to-death of children was 26 months.

Overall, the prevalence of experiencing child’s death rate in the Tigray Regional State was 4.2%.

The multivariable Cox regression model analysis showed that living rural place of residence (AHR = 19.8; 95% CI: (7.25–54.049)), being unvaccinated child (no) (AHR = 2.76; 95% CI: (1.071–7.11)), and poor wealth index (AHR = 15.4; 95% CI: (2.83–84)) were statistically significant predictors of time-to-death rate of children’s.

**Conclusion:**

The study recognized that being a rural place of residence, unvaccinated child status (no) and poor wealth index were statistically significant predictors of children’s short time-to-death rate.

## Background

The under-five mortality rate is defined as the probability of dying between birth and exactly 5 years of age, expressed per 1000 live births, and it is an influential indicator that is statistically related to the well-being of people. It is important as one of the progression indicators of healthiness, socio-economic levels, and quality of life of certain residents [1, 2].

Approximately 5642 million children under 5 years of age have died worldwide since 2016. Of these, 2860(50.7%) deaths occur in sub-Saharan African countries, including Ethiopia [1].The mortality rate of children is 5 times greater in developing countries than in developed countries [3].

In 2015, several countries worldwide decided to alleviate the death rate children by two-thirds of the Millennium Development Goal. According to the report of 2001, the United Nations prepared progression towards children’s existence goals [4].

The mortality rate of under 5 years old children is still the main community serious challenging issue of public health concern. Even though Ethiopia showed a substantial reduction in under-five mortality over time but the rate of reduction is low among regions including Tigray Regional State [5].

Even though there are vertical problems, some countries have a higher risk of a child’s death rate stated since 1990, which is illustrated as odds, indicating that progression for entirely children is inside our understanding. Similarly, Bangladesh and Liberia succeeded in decreasing the below-5-year-old death rate by at least 66.7% in 1990. For example, Ethiopia, Malawi, Madagascar, and Niger in the sub-Saharan Africa (SSA) and Nepal and Bhutan in Southern Asia had showed decreases of at least 60%. Several researchers found that the death rate, of children below 5 years old in Ethiopia has been decreasing [5–8].

There are several reasons to decrease in these studies. The main decreasing rates of the studies are agriculture in the nationwide economy, the growth of urbanization, and the initiation of globalization, which has faster economic growth in the country [9]. Similarly, mother’s education, mother’s age, place of residence, wealth index and marital status are predictor factors of the child mortality rate. These factors are associated with women’s autonomy, healthiness on the look-out for conduct and other predictor factors that have a higher hazard rate of mortality children’s existence merit of additional studies such that interferences could be planned to stand-in alleviations in children’s rate of mortality by allowing for the desires and wellbeing of women, including the essential factors for mother’s education and socioeconomic welfare [2, 6, 10–12].

Furthermore, the reasons for the large number of children under 5 years old mortality rates are that poor resource surroundings are difficult and merit concentrated efforts to illuminate their consequences to increase children’s survival time [11]. Today, the world is not yet on track to accomplish the Millennium Development Goals role objective of 66.7% to decrease the mortality rate of children in 2015. Several researchers and demographers recommend conducting in-depth investigations on the different characteristics of children’s healthiness status in various sociodemographic and economic settings. Investigators have previously recorded an essential study on, sociodemographic, economic, health and environmental predictor factors of the death rate of under 5 years of age children in Ethiopia [13].

The Ethiopian government executed the Health Extension Program and provided that comprehensive, impartial and reasonable health care for the rural population to promote, protective and basic medicinal services since 2003 [14]. For this reason, the 2016 Ethiopia Demographic and Health Survey (EDHS) report indicate an overall decrease in the under-5 year- old death rate from 166/1000 in 2000 to 67 deaths/1000 births 2016. However, the Tigray Region State is experiencing a greater risk of mortality rate of 85 deaths/1000 births compared to the risk of mortality national level average rate of 67 deaths/1000 births in 2016 [15]. Moreover, several researchers are focused on the national level. Such investigations have paucity of information on policy makers and implementers to plan and design appropriate intervention strategies to prevent the associated mortality rate among children under 5 years of age, as the national effects might not indicate the precise situation at regional levels. To solve this knowledge gap, the authors conducted a comprehensive retrospective cross sectional study design analysis on the current 2016 EDHS dataset.

### Objective of the study

#### General objective

The general objective of this current investigation was to identify the major predictor factors that shorten the time-to-death rate of children in the Tigray Regional State, Ethiopia.

#### Specific objectives

- ➢ To identify socio demographic, economic and clinical predictor factors associated with time-to-death rate of children.
- ➢ To estimate the survival time and compare survival curves between the different category predictor factors associated with time-to-death rate of children.

## Methods

### Study design, period, data sources, and setting

This investigation was carried out based on evidence of secondary data analysis of a cross-sectional retrospective study design that took place 5.5 months from 18 January 2016 to 27 June, 2016 of EDHS in Ethiopia. The survey was conducted by the Central Statistics Agency, the Ministry of Health and the Ethiopian Public Health Institute. Their source was funded by the United States Agency for International Development (USAID). The raw data were found from the Demographic and Health Survey measure Program with agreement [16]. The main aim of this information under-5 mortality is significant to demographic assessment of population, socioeconomic growth, quality of life, support to estimate how many children might be at greater risk of death, and help an improvement of strategies to reduce this higher risk.

### Study participants, inclusion and exclusion criteria, and sample size

In this investigation all live births in the 5 years born women are who’s aged (15–49 years) were involved in the Tigray Regional State on the 2016 EDHS. Those women and who were either stable resident of the chosen households, or who lived in the household at least one night before the survey, were eligible for the interview. Data were collected by face-to-face interviewing women that met the eligibility criteria. Moreover, full records of all children under-5 years old were included in the study. However, children with incomplete records were excluded. In the Tigray Regional State 1033 births were reported in the last 5 years preceding the survey. Next, child with information on death during the last 5 years were identified. Finally, a total of 1018 children that have completed information about all the predictor factors considered were included in the study.

### Study variables

The dependent (response) variable is children’s time-to-death, divided into death and censored events, which were measured in the months. Censored denotes actively surviving children. In this study, the time gap measured (in month) starting from the child birth date until the occurrence of interested outcome of observation (death/censored) was reached.

The independent variables included in this study were: mother’s education level (0 = higher,1 = secondary,2 = primary, 3 = no education), mother’s age (discrete), place of residence (0 = urban,1 = rural),water drink source (0 = protected water,1 = unprotected water), wealth index combined income of households (0 = rich,1 = middle, 2 = poor), mother’s marital status (0 = married,1 = divorced/widowed,2 = single), child vaccination status (0 = yes,1 = no), gender of child’s status (0 = female,1 = male).

The authors defined and coded the independent variable wealth index combined income of households as follows: 0 = rich (include richer and richest), 1 = middle, and 2 = poor (includes poorest and poor).These 2 categories have composited because the frequency distribution of events in the poorest and richest categories is too small.

### Data quality and controlling bias

The data for the original work had collected by using comprehensive standard questionnaires.

The woman’s questionnaire, the household questionnaire, and the man’s questionnaire were employed to gather economic and socio-demographic information and other essential information’s. After all the questionnaires were prepared in English, they were translated into Tigrigna language. In addition to this, the gathered data was back translated into English to keep consistence. The quality of the dataset had maintained by testing its completeness, handling the missing values by running frequency tables. Moreover, the quality of the original dataset were maintained by pretest of the questionnaires in Tigrigna language, giving training for interviewers and interviewers used tablet computers to register the response.

### Statistical data analysis

The collected data were entered into Excel of CSV format, coded, cleaned, and analyzed by using statistical software R-version 3.6.1. The study used descriptive statistics such as frequency distributions and percentages to define the sample information. The Kaplan-Meier (K-M) survival curve was used to compare the survival probability of categorical variables of under-five year’s age of children.

One of the most common types of regression models used in survival analysis is the Cox proportional hazard model. Moreover, this model has a significant role in the investigation of the survival time-to-death of children under-five years of age by estimating hazard ratio (HRs) and with 95% confidence intervals (CIs).

Cox proportional hazard model, $$ {\begin{array}{c}.\\ {}h(t)=h\end{array}}_0(t)\ast {\mathit{\exp}}^{\left({b}_1{x}_1+\kern0.5em {b}_2{x}_2\kern0.5em +\kern0.5em {b}_3{x}_3+\dots \dots \dots \dots ..\kern0.5em {b}_p{x}_p\right).} $$

The hazard function *h*(*t*) is identified by the set of p covariates (*x*_1_, *x*_2_, *x*_3_,……, *x*_*p*_), whose effect is measured by the magnitude of the corresponding coefficients (*b*_1_, *b*_2_, *b*_3_,……, *b*_*p*_). The term *h*_0_ (t) is called the baseline hazard for a survival time-to-death [17, 18].

## Results

### Descriptive statistics

The main sociodemographic, economic and clinical variables of the participants with a 5 year death of rate child are described in Table 1. The total number of children included in the current study was 1018. Of these children, 43(4.2%) were dead whereas 975(95.8%) were alive at the date of the data collection process. That means 1 in 24 children in the Tigray Region State die before reaching 5 years old. The death rate of children varied based on the type of place residence; a greater rate of death (2.4%) was recorded in rural areas than in urban areas (1.9%).

**Table 1:** Summary of sociodemographic, economic and clinical predictors of death rate under-five years children in the Tigray Regional State, Ethiopia, from 18 January 2016 to 27 June 2016 (*N* = 1018)

		Status of under-five years children		
Variables	Categories	Censored N (%)	Death N (%)	Total
Wealth index combined	Rich	310(30.5%)	3(0.3%)	313(30.8%)
	Middle	145(14.2%)	1(0.1%)	146(14.3%)
	Poor	520(51.1%)	39(3.8%)	559(54.9%)
Mother’s education level	Higher	23(2.3%)	1(0.1%)	24(2.4%)
	Secondary	99(9.7%)	2(0.2%)	101(9.9%)
	Primary	314(30.8%)	6(0.6%)	320(31.4%)
	No Education	539(53%)	34(3.3%)	573(56.3%)
Place of residence	Urban	165(16.2%)	19(1.9%)	184(18.1%)
	Rural	810(79.6%)	24(2.4%)	834(82%)
Water drink source	Protected Water	378(37.1%)	8(0.9%)	386(38%)
	Unprotected Water	597(58.6%)	35(3.4%)	632(62%)
Mother’s marital status	Married	870(85.5%)	38(3.7%)	908(89.2%)
	Divorced/Widowed	95(9.3%)	3(0.3%)	98(9.6%)
	Single	10(1%)	2(0.2%)	12(1.2%)
Child vaccination status	Yes	671(65.9%)	8(0.8%)	679(66.7%)
	No	304(29.9%)	35(3.4%)	339(33.3%)
Gender of child status	Female	494(48.5%)	15(1.5%)	509(50%)
	Male	481(47.2%)	28(2.8%)	509(50%)

The rate of mortality of children varies by wealth index combined income of households. By wealth index combined income of households, the highest rate of death of children was observed between poor mothers’ economic statuses (3.8%) when compared to children living among rich mothers in economic status (0.3%).

Similarly, a higher mortality rate of children was observed in non-vaccinated children (3.4%) as compared to vaccinated children (0.8%), and taking protected sources of drinking water can reduce the different deaths rate of children in number. Children using an unprotected source of drinking water had a higher death rate of children (3.4%) than using a protected source of drinking water (0.9%) (See Table 1).

**Fig. 1:**
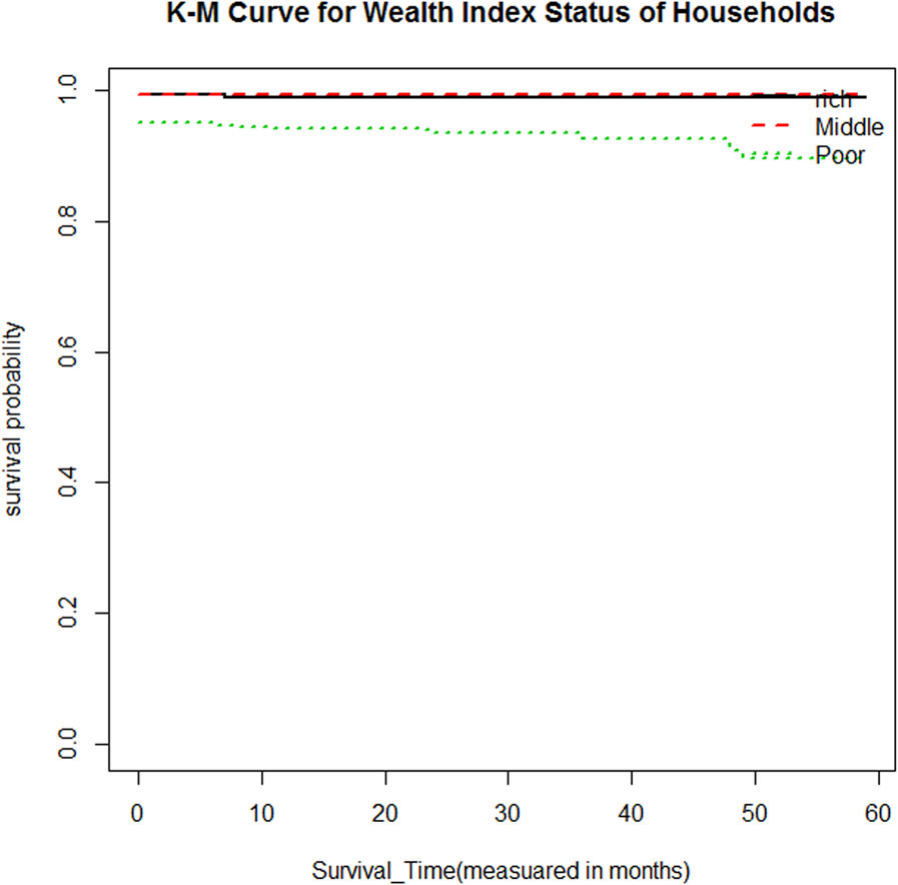
Survival curve by water drink source

**Fig. 2:**
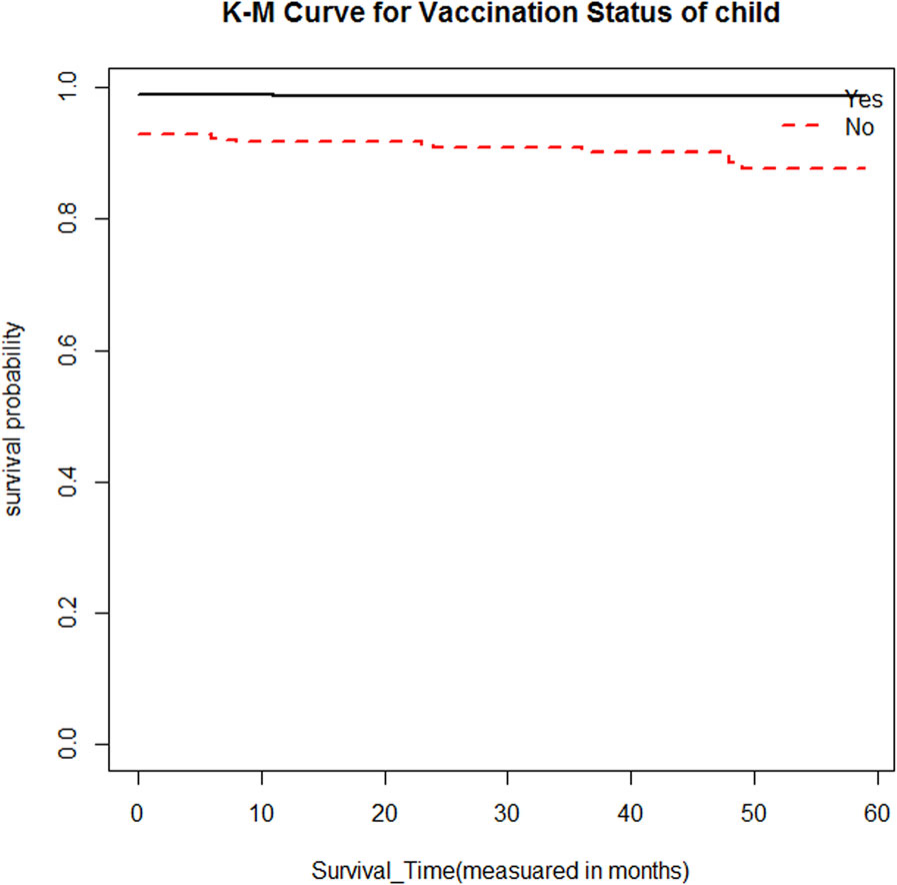
Survival curve by residence differentials place

**Fig. 3:**
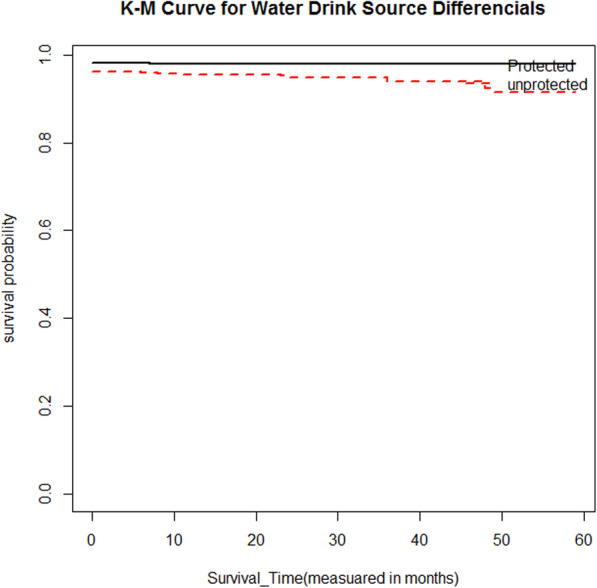
Survival curve of wealth index combined status

**Fig. 4:**
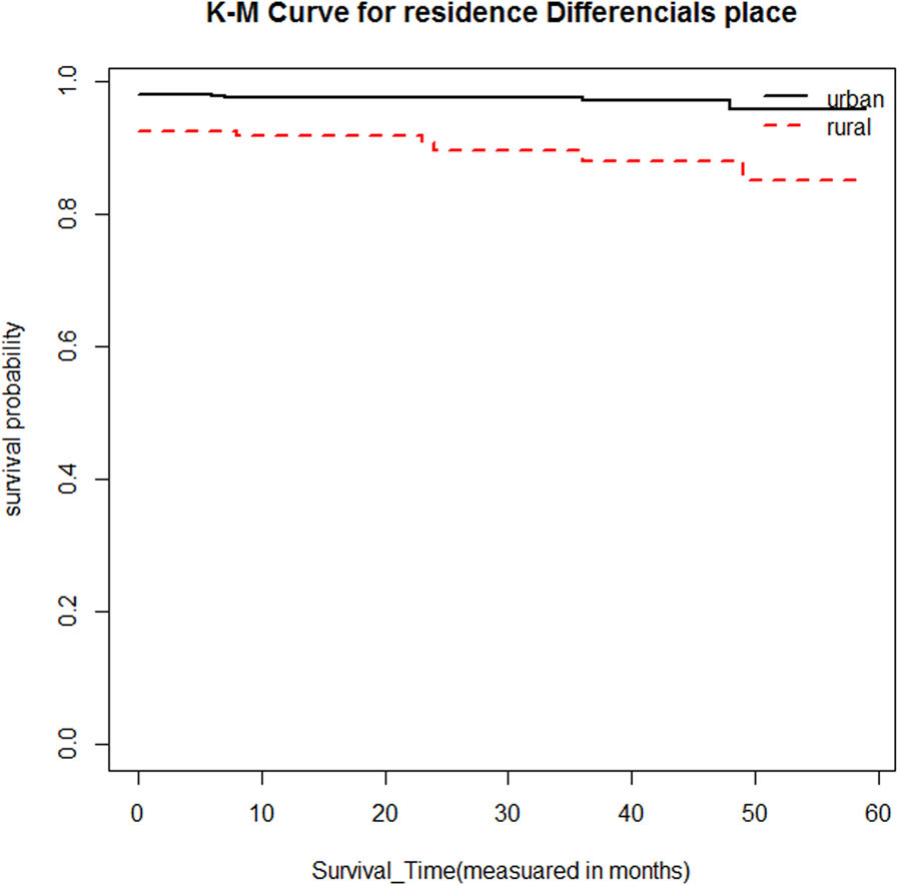
Survival curve of vaccination status of child

Moreover, to the descriptive statistics given in Table 1, Kaplan-Meier (K-M) survival curve estimators are plotted for most essential predictor variables (Fig. 1, 2, 3, 4). These sample Kaplan-Meier (K-M) curves show that under-five years old children who live in rural areas, use unprotected water drink sources, vaccination status of child (no), and poor wealth index status of households have a short survival time compared to those reference categories.

### Predictors of time-to-death under-five years old of children

In crude hazard ratio (CHR), the result indicated that poor wealth index of household combined, rural place of residence, and child vaccination status(no) were statistically significant predictors of death rate of children’s (*P*-value < 0.05). Children who don’t vaccinate are higher risk of death rate than vaccinated children (CHR = 7.65, 95% CI: 3.53–16.6). Children whose mothers were poor wealth index combined income of households (CHR = 7.24, 95% CI: 0.073–6.74) were at greater risk of death rate than children whose mothers were rich wealth index combined income of households. Children who live in rural areas also had a higher risk of death than children who live in urban areas (CHR = 3.66, 95% CI: 2.004–6.68) (See Table 2).

**Table 2:** Bivariable and multivariable Cox regression analysis of sociodemographic, economic and clinical predictors associated with under-five death rate of children in the Tigray Regional State, Ethiopia, 2016 (*N* = 1018)

		95% CI for HR				95% CI for HR		
Variables	CHR	Lower	Upper	P-value	AHR	Lower	Upper	P-value
Wealth index combined(ref. = rich)								
Middle	0.7	0.073	6.74	0.760	1.52	0.104	22.2	0.80
Poor	7.24	0.073	6.74	0.0009^a^	15.4	2.83	84	0.0016^a^
Mother’s education level(ref. = higher)								
Secondary	0.47	0.043	5.21	0.541	0.041	0.002	1.077	0.06
Primary	0.44	0.052	3.62	0.441	0.578	0.049	6.875	0.67
No education	1.35	0.185	9.88	0.766	1.527	0.126	18.586	0.74
Water drink source(ref. = protected water)								
Unprotected water	2.7	1.24	5.8	0.012 ^a^	2.24	0.94	5.6	0.07
Place of residence(ref. = urban)								
Rural	3.659	2.004	6.68	0.0000^a^	19.8	7.25	54.049	0.0000^a^
Mother’s marital status(ref. = married)								
Divorced/Widowed	0.67	0.207	2.18	0.507	0.246	0.047	1.2791	0.095
Single	3.84	0.925	15.9	0.064	0.333	0.032	3.505	0.36
Gender of child status(ref. = female)								
Male	1.84	0.98	3.44	0.0573	0.954	0.460	1.978	0.90
Mother’s age	1.01	0.97	1.06	0.553	1.07	0.994	1.1519	0.07
Child vaccination status (ref. = yes)								
No	7.65	3.53	16.6	0.0000^a^	2.76	1.071	7.11	0.035^a^

*CHR* crude hazard ratio, *AHR* adjusted hazard ratio,^a^ Significant at 5% level of significance, *ref* reference, *CI* confidence interval

From the adjusted hazard ratio (AHR), the results showed that poor wealth index combined income of households, rural place of residence, and child vaccination status (no) were statistically significant predictors of death rate of children’s (*P*-value < 0.05). The adjusted hazard ratio (AHR) of the death rate was 15.4 times higher among children who had a poor wealth index combined income of households than among those who were rich (AHR = 15.4; 95% CI: (2.83–84)). When we compare place of residence, we found statistically significant predictors of the child mortality rate. The AHR of the child death rate was approximately 19.8 times higher among children in rural areas than among urban residents (AHR = 19.8; 95% CI: (7.25–54.049)). There was no significant difference in the risk of child death rate between the sex child’s status of females and males. Moreover, for the variable vaccination status of Children, the AHR of child mortality of unvaccinated children was approximately 2.76 times higher than that of children who had vaccinated status of Children (AHR = 2.76; 95% CI: (1.07–7.11)) (See Table 2).

The result of likelihood, Wald, and score test had a *p*-value = 0.000, which is statistically significant at the 5% level of significance and the concordance = 0.9 (se = 0.03) illustrated that the data and the model were in good fit. Further, since the overall Global test (p-value = 0.70) for all predictors are > 0.05 and none of the predictor variable was failed Cox proportional hazard model the necessary assumption. Therefore, model violated the Cox proportionality assumptions and it is mathematically adequate (See Table 3).

**Table 3:** Test of proportional-hazards assumption (R version 3.6.1)

Predictor variable	Chisq	Df	Prob>Chisq
Wealth index combined	0.74	2	0.69
Mother’s age	0.07	1	0.79
Mother’s education level	0.88	3	0.83
Water drink source	0.9	1	0.34
Place of residence	2.2	1	0.14
Mother’s marital status	2.5	2	0.28
Gender of child status	0.0022	1	0.96
Child vaccination status	0.62	1	0.43
Global test	9	12	0.70

*N.B* chisq refers to chi-square statistic value, DF refers to degree of freedom, Prob>Chisq refers to P-value

## Discussion

The study empirically examined and recognized the predictor factors that were related to the mortality rate of child in the Tigray Regional State using the EDHS data. Both descriptive statistics and multivariable Cox regression model analysis were employed to analyze the secondary data.

The combined household wealth index was an essential socioeconomic variable that predicts the death rate of children in the Tigray Regional State. Childs born from the poor wealth index combined income of households group were obtained, at a greater hazard of being dying earlier celebrating their first week childbirths than children from the rich wealth index combined income of households. Previous literature showed that mothers’ economic level had better access to rich currency incomes than poor wealth, permitting them an excellent diet and better access to medical treatment [19, 20].

Place of residence was another statistically significant predictor of the death rate of children, such that children from rural areas had a greater risk of death than children from urban parts. Previous studies furthermore stated that urban residents had a better wealth index, better access to hygiene facilities, better healthiness care facilities and media access for healthiness facility utilization for their new birth [12, 21, 22].

The accurate vaccination status of children was also a key factor significantly associated with the death rate of children in the Tigray Region State. If children were not vaccinated for a long time, they were more likely to die in the early newborn period than those who vaccinated on the proper time. This is because of other infectious diseases such as polio and intestinal parasites. Such a result is also found in Southwest Ethiopia [23]. In such case, health services providers should be given special attention to aware mothers for treating their child vaccination status. Finally, community-based longitudinal investigations will be more useful to obtained further unmeasured risk predictors.

### Limitations and strengths of the study

This investigation considered national state representative survey data that improve inferences for the whole country level. Nevertheless, this investigation used secondary data, and the main limitation of this investigation was dependent on a mother’s recall events that were conducted for the previous 5 years prior to the survey dataset, which is subject to remembrance bias. Perhaps this might be because mothers (females) are frightened to report fetal deaths due to certain beliefs and traditions in the people. Further, the authors were unable to include some of the important predictor variables which have been identified as associated factors due to the reason of high rates of missing data for these variables. As a result, the associated factors studied were a convenience sample of data gathered as part of the DHS and it’s not clear how informative these results are (given the omission of some of the historically significant data).

## Conclusion

This investigation was carried-out to identify the major associated factors of death rate of children in the Tigray Regional State. The multivariable Cox regression model analysis showed that being a rural place of residence, unvaccinated child status and having a poor wealth index were statistically significant predictors of a short survival time-to-death rate of children.

## Data Availability

The dataset will be obtainable based on request from the corresponding author on reasonable request.

## Abbreviations

AHR: Adjusted Hazard ratio
CHR: Crude Hazard ratio
CI: Confidence Interval
EDHS: Ethiopian Demographic and Health Survey
K-M: Kaplan Meier
Ref: Reference
SSA: Sub-Saharan African
USAID: United States Agency for International Development
